# Population Pharmacokinetics of Subcutaneous Infliximab: A Real-World Study of Naïve and Switch Patients

**DOI:** 10.3390/pharmaceutics18091162

**Published:** 2026-09-15

**Authors:** Blanca Vicente, Hinojal Zazo, José Germán Sánchez-Hernández, Natalia Revilla, Paulo Teixeira-da-Silva

**Affiliations:** 1 Pharmacy Service, University Hospital of Salamanca, 37007 Salamanca, Spain; bvicenteg@saludcastillayleon.es (B.V.); jgermansanchez@saludcastillayleon.es (J.G.S.-H.); 2 Area of Pharmacy and Pharmaceutical Technology, Pharmaceutical Sciences Department, Universidad de Salamanca, 37007 Salamanca, Spain; paulo@usal.es; 3 Institute of Biomedical Research of Salamanca (IBSAL), 37007 Salamanca, Spain; 4 Pharmaceutical Pharmacy Service, University Hospital of Burgos, 09006 Burgos, Spain; nrevillac@saludcastillayleon.es

**Keywords:** subcutaneous infliximab, pharmacokinetics, therapeutic drug monitoring (TDM), inflammatory bowel disease (IBD)

## Abstract

**Background**: Subcutaneous infliximab (SC-IFX) represents a significant advance for patients with inflammatory bowel disease (IBD) due to the possibility of home self-administration. However, at this time, there are no clear recommendations for implementing model-informed precision dosing (MIPD) in accordance with real-world clinical knowledge. **Objective**: The aim of the present study is to develop a population pharmacokinetic (PopPK) model for SC-IFX. **Methods**: A multicenter retrospective observational study was performed in adult patients (both naïve patients and those switched from intravenous administration) diagnosed with IBD. PopPK analysis was performed using NONMEM with a nonlinear mixed-effects model approach. A stepwise covariate modeling approach was implemented to identify anthropometric and clinical factors influencing SC-IFX disposition. The bootstrap method was used for internal model validation. **Results**: A total of 250 serum concentration samples of IFX, corresponding to 45 patients, were analyzed. A one-compartment model with first-order absorption adequately described the data. The final model included total body weight and suspected immunogenicity as covariates of clearance (CL). Suspected immunogenicity was associated with a 70.0% increase in clearance, while each kg deviation from the median weight (73 kg) modified clearance by 0.888%/kg. **Conclusions**: A PopPK model for SC-IFX was successfully developed and validated using European real-world data. This exploratory model establishes a foundation for future MIPD applications, offering a structured approach to initiate or transition patients to SC-IFX therapy.

## 1. Introduction

Inflammatory bowel disease (IBD), encompassing ulcerative colitis (UC) and Crohn’s disease (CD), is a chronic immune-mediated condition characterized by progressive gastrointestinal damage. Infliximab (IFX), a chimeric monoclonal anti-tumor necrosis factor-alpha (anti-TNF) antibody, remains a cornerstone of first-line biological therapy [1].

For many years, the intravenous (IV) formulation has been the standard of care. However, the recent approval of subcutaneous infliximab (SC-IFX) for maintenance therapy represents a significant shift in the treatment paradigm. This development offers increased patient autonomy and improved healthcare resource utilization [2,3,4]. Nevertheless, information regarding the pharmacokinetics (PK) of SC-IFX remains limited.

Clinical trials have demonstrated the non-inferiority of SC-IFX (at a fixed dose of 120 mg every 2 weeks) compared to IV-IFX (5 mg/kg every 8 weeks) regarding efficacy, safety, and immunogenicity [4,5]. Standard SC-IFX dosing regimens were validated in controlled environments using fixed IV induction schedules. In real-world clinical practice, a substantial proportion of patients undergo dose intensification (either by increasing the dose or shortening the interval) guided by Therapeutic Drug Monitoring (TDM) to overcome suboptimal responses [6].

The exposure–response relationship of IFX is well established for the IV route during maintenance therapy. Target trough concentrations (C_min_) are recommended to range from 3 to 5 mg/L for clinical remission and 7 to 10 mg/L for mucosal healing [6,7,8]. In contrast, the optimal PK targets for SC-IFX are still being defined. The transition to SC administration leads to a flatter serum concentration profile, characterized by considerably higher steady-state C_min_ and reduced peak-to-trough fluctuations. Therefore, it is not possible to directly extrapolate standard IV C_min_ targets to SC-IFX, as emerging evidence indicates that higher trough thresholds are required [5,6,9].

Despite its potential, there is a lack of evidence-based guidelines on how to integrate TDM during the IV-to-SC switch, especially in patients previously requiring intensified IV regimens. For this reason, the aim of the present study is to develop a population pharmacokinetic (PopPK) model for SC formulation, based on the pharmacokinetic characteristics of a real-world database of adults with IBD who received SC-IFX.

## 2. Materials and Methods

### 2.1. Study Design and Patient Population

This multicenter, retrospective, observational study was conducted in a real-world clinical setting at two centers in Spain: the University Hospital of Burgos and the University Hospital of Salamanca. The study was approved by the Ethics Committee for Clinical Research with Medicines of the Burgos-Soria Area (Ref. CElm 2711) and the Ethics Committee for Clinical Research with Medicines of the Salamanca Health Area (Ref. CEIm: PI 2024 05 1682) after evaluating compliance with ethical standards and good clinical practices.

Data were extracted from electronic medical records of routine clinical monitoring procedures and outpatient records of the pharmacy departments. The inclusion criteria were adult patients diagnosed with IBD, either CD or UC, who received SC-IFX between January 2020 and June 2024. The cohort included both anti-TNF naïve patients (initiating IFX via the SC route after IV induction) and switch patients (transitioning from IV-IFX maintenance therapy to SC-IFX). Patients were excluded if treatment adherence was <80%, as estimated by pharmacy dispensing records for SC-IFX and infusion logs for IV-IFX.

The standard SC-IFX regimen was 120 mg administered every 2 weeks. However, alternative dosing intervals (e.g., weekly, every 10 days, or every 3 weeks) were used for some patients to achieve C_min_ concentrations generally within the 12–20 mg/L range, depending on the patient’s clinical status and therapeutic goals, in accordance with the limited exposure–response evidence available for SC-IFX [5,9,10]. The IV-IFX dosing regimens administered ranged from 5 to 10 mg/kg at intervals of 4 to 8 weeks. The IV dose adjustments were based on clinical response, biomarkers (e.g., fecal calprotectin), and TDM. During maintenance, IV C_min_ targets were individualized according to the therapeutic endpoint. Concentrations around 3 mg/L were considered adequate for clinical response, while higher concentrations (approximately 7–10 mg/L) were considered appropriate for more stringent outcomes, such as endoscopic healing. This approach aligns with established exposure–response evidence and current TDM recommendations for IV-IFX [6,7,8].

### 2.2. Analytical Procedures and Immunogenicity Assessment

Serum samples were collected immediately prior to administration, C_min_, and during induction (weeks 2, 6, and 14) and maintenance (every 6 months or more frequently if clinical loss of response occurred). Serum IFX concentrations and anti-drug antibodies (ADAs) were measured using a validated drug-sensitive capture enzyme-linked immunosorbent assay (ELISA; Promonitor^®^, Grifols, San Diego, CA, USA). The ELISA assay is drug-sensitive, meaning that the presence of circulating drugs could potentially interfere with ADA detection, thereby underestimating the true prevalence of immunogenicity. Consequently, an exploratory, observation-level binary covariate for “suspected immunogenicity” (SUSP) was defined. Individual concentration observations were coded as SUSP-positive if they met any of the following criteria: (i) analytical confirmation of ADAs or (ii) a decrease in C_min_ of more than 33% compared with the previous stable level. It is important to note that these observations could not be explained by other potential factors, such as non-adherence, recent dose changes, delayed administration, or acute flares. Acute flares were assessed based on clinical evaluation and stable C-reactive protein (CRP) and fecal calprotectin (FCP) values, which were measured at each TDM sampling visit [11]. SUSP was implemented as a time-varying covariate and should be interpreted as a PK-informed surrogate marker rather than as confirmed immunogenicity.

### 2.3. Population Pharmacokinetic Modeling

Based on prior knowledge and data sparsity, a one-compartment model with first-order absorption and elimination was implemented (ADVAN2, TRANS2). The model was parameterized in terms of subcutaneous bioavailability (F_SC, estimated on the logit scale), clearance (CL), the volume of distribution (V), and the first-order absorption rate constant (k_a_).

Due to the nature of the database, which exclusively contains C_min_, there is a possibility that the estimation of Ka may be inaccurate. Therefore, a sensitivity analysis was performed on the Ka value. The values tested were 50%, 75%, 125%, and 150% of the pivotal study’s estimated value (0.0113 h^−1^) [12]. All other estimable model parameters were re-estimated for each scenario. The robustness of the model was assessed by comparing several key metrics. These included parameter estimates, relative standard errors, variabilities, and covariate effects.

Inter-individual variability (IIV) was modeled using an exponential error structure, assuming a log-normal distribution. Residual unexplained variability (RUV) was evaluated using additive, proportional, and combined error models.

A stepwise covariate modeling (SCM) approach was implemented. Potential covariates were selected based on clinical relevance and previous reports:

- Continuous covariates: age, weight (TBW), height (HGT), body mass index (BMI), FCP, serum creatinine, serum albumin (ALB), ferritin, hemoglobin, and CRP.
- Categorical covariates: sex, diagnosis (CD vs. UC), administration route (IV vs. SC), previous IFX treatment status (STAT) (naïve or switch), prior biological exposure, concomitant immunosuppression (thiopurines/methotrexate), and immunogenicity (ADA and SUSP).

The SCM process involved forward inclusion (*p* < 0.05, ∆OFV > 3.84) followed by backward elimination (*p* < 0.01, ∆OFV > 6.63) to ensure the inclusion of only statistically significant and clinically parsimonious predictors [13,14].

### 2.4. Model Evaluation

The model’s fit to the data was evaluated based on the following criteria: successful convergence, stability of parameter estimates, and a significant reduction in the objective function value (OFV). The internal validation process was conducted using the following methodology [15,16]:

- Goodness-of-fit (GOF) diagnostic plots.
- Bootstrap analysis (*n* = 1000 resamples) to determine the 95% confidence intervals (CIs) of the parameters. Relative error was calculated as 100× ((bootstrap median)—(final-model estimate))/(final-model estimate).
- Visual predictive checks (VPCs) with dose-normalized data to assess the model’s ability to capture the central tendency and variability of the observed concentrations over time.

### 2.5. Software

PopPK analysis was performed using nonlinear mixed-effects modeling (NONMEM v.7.5.1). Parameters were estimated using the first-order conditional estimation method with interaction (FOCEI). Data management and visual diagnostics were conducted in R (v.4.3.2) and PsN (v.5.3.0).

## 3. Results

### 3.1. Study Population

A total of 45 adult patients with IBD were included in the PopPK analysis. The final dataset comprised 250 serum IFX concentrations, with a median of 6 (IQR 4–7) samples per patient. All patients demonstrated high treatment adherence (>80%) and were included in the model development.

At the time of sampling, both IV and SC-IFX were administered following TDM-guided intensified or standard regimens. Among the 29 switched patients, only 34% were on the standard guideline-recommended IV maintenance dose, whereas the remaining patients had undergone dose intensification based on TDM. In the context of the SC-IFX treatment, a TDM-guided regimen was administered to a mere six patients (13.3%) from the overall cohort (*n* = 45), encompassing both naïve and switch patients.

Baseline demographic and clinical characteristics, including laboratory parameters, are summarized in Table 1. In this table, immunogenicity is outlined according to patient count; at the sample level, ADA and SUSP positivity were identified in 4 out of 138 (2.9%) and 32 out of 250 (12.8%) samples, respectively, highlighting a substantial lack of data for ADA, with 112 samples (44.8%) remaining unevaluated.

#### Observed Infliximab Concentrations

The analysis of observed serum IFX C_min_ revealed an exposure profile across the study population receiving SC-IFX. The median steady-state C_min_ for the entire cohort was 14.3 mg/L (IQR: [10.0–16.6]). When stratifying the population, no statistically significant differences were observed in C_min_ between naïve and switch patients (Table 2).

### 3.2. Population Pharmacokinetic Analysis

#### 3.2.1. Model Development

A one-compartment structural model with first-order absorption and elimination adequately described the pharmacokinetic profile of SC-IFX. Following a sensitivity analysis of Ka (see Table S1), the value was set to 0.27 d^−1^ (0.0113 h^−1^), aligning with the estimation derived from the pivotal SC-IFX population model [12]. For the same reason, the IIV of V and F_SC was fixed at 0% to ensure model stability [17], while a proportional error model was used to describe RUV. The variability shrinkage was lower than 8%, and the PK parameters were estimated with six significant digits. The covariate analysis revealed a statistically significant impact of TBW and SUSP on CL, resulting in a decline in the objective function value (OFV) of 35.54 points. The inclusion of these covariates is described by the following equation in the final model:

CL [L/h] = 0.017 × (1 + 0.70 × SUSP) × (1 + 0.00888 × (TBW − 73))(1)

where SUSP is a binary variable (1 if present and 0 if absent), and TBW is centered on the population median of 73 kg.

According to this model, observations classified as suspected immunogenicity (SUSP = 1) were associated with a 70.0% higher CL, while each 1 kg deviation from the median TBW modified clearance by 0.888%/kg. In order to illustrate the magnitude of the TBW effect under fixed-dose SC administration, post hoc simulations were performed for TBW corresponding to the 25th, 50th, and 75th percentiles of the study population (Figure S1).

#### 3.2.2. Model Evaluation

**Figure 1:**
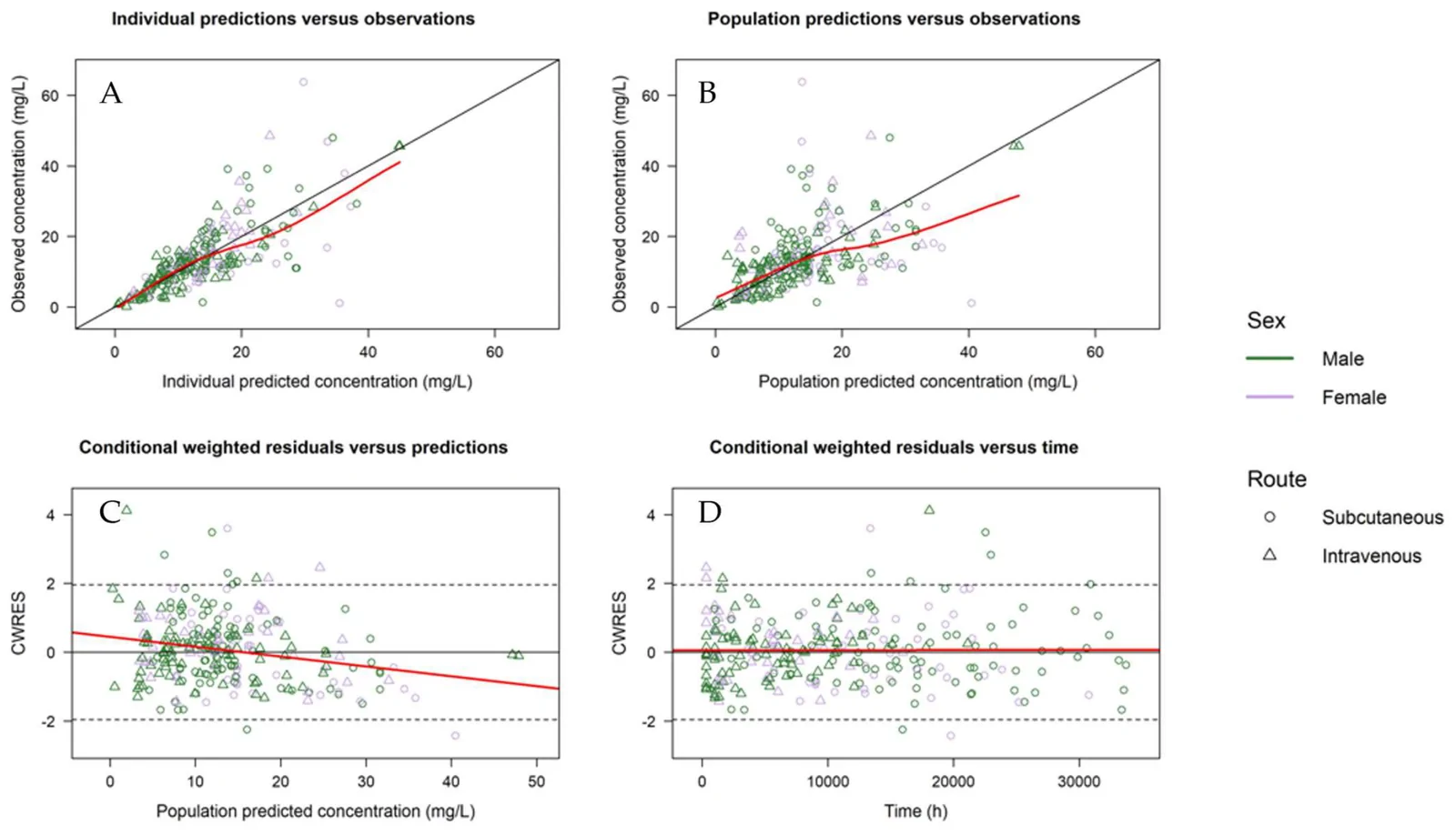
Goodness of fit for the pharmacokinetic model. Observed concentrations are plotted against individual predictions (**A**) and population predictions (**B**). Conditional weighted residuals (CWRESs) are shown against population predictions (**C**) and against time (**D**). Men are shown in green and women in purple; circles indicate subcutaneous administration, and triangles indicate intravenous administration. The red line represents the LOESS smoothing of the data. In the upper panels, the solid black line corresponds to the identity line; in the lower panels, the solid black line indicates CWRES = 0, and the dashed lines indicate the reference limits of ±1.96.

The final model demonstrated good predictive performance and stability. GOF plots (Figure 1A,B) showed an excellent correlation between the observed and predicted concentrations, with no significant bias. Conditional weighted residuals (CWRESs) were randomly distributed around zero (Figure 1C,D), and diagnostic plots stratified by sex confirmed that the model performed consistently despite the gender imbalance in the cohort.

Internal validation using bootstrap analysis confirmed the precision of the parameter estimates (Table 3), with 100% of bootstrap samples being able to converge successfully.

**Figure 2:**
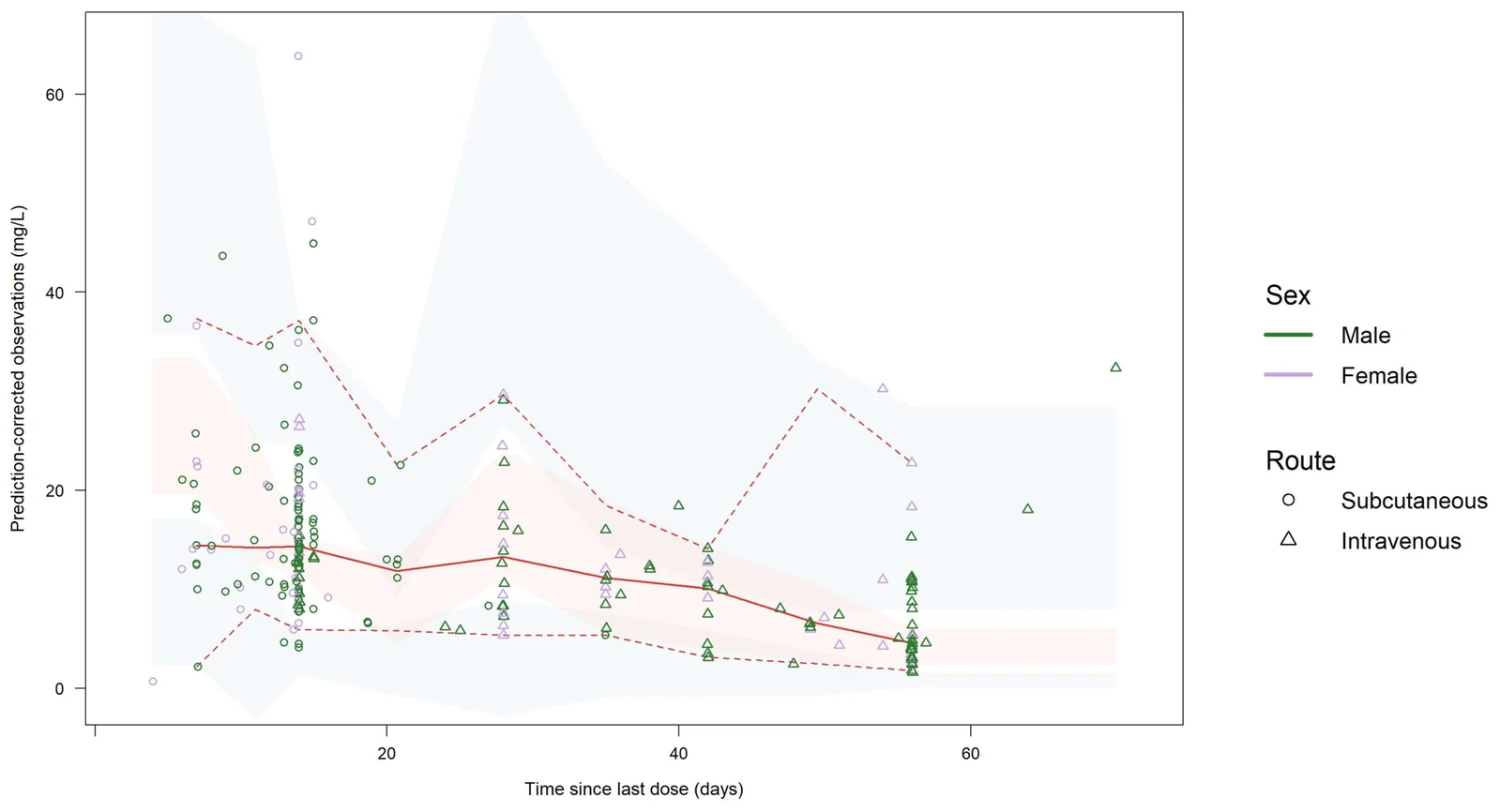
Prediction-corrected visual predictive check of the final population pharmacokinetic model. Green and purple represent prediction-corrected observations in male and female participants, respectively; circles indicate subcutaneous administration, and triangles indicate intravenous administration. The solid red line represents the median of the observed data, whereas the dashed red lines represent the 10th and 90th percentiles of the observed data. The red shaded area represents the 95% confidence interval of the simulated median, and the blue shaded areas represent the 95% confidence intervals of the simulated 10th and 90th percentiles.

The relative bias between the final model parameters and the bootstrap medians was lower than the 10%, and all original estimates fell within the 95% CI of the bootstrap results. The VPC (Figure 2) further confirmed the model’s accuracy. The majority of the observed data points were contained within the 90% prediction interval, and the predicted median closely tracked the observed central tendency, indicating that the model captured both the average pharmacokinetic behavior and the variability of SC-IFX in this real-world population.

## 4. Discussion

This study addresses a critical gap in the literature by evaluating the “real-life” PK performance of a uniform 120 mg SC dose and developing a PopPK model for SC-IFX, although based on a modest sample size. The database was constructed based on a real-world cohort encompassing both naïve and switch patients. Furthermore, in line with real clinical practice, some of our switch patients underwent dose intensification during their prior IV maintenance phase (up to 10 mg/kg or at 4-week intervals). Unlike the standardized, weight-based IV regimens and controlled environments of pivotal trials [4,5], our data reflect the PK of the complex transition from IV to SC administration in a "real-world" clinical setting.

In line with pivotal trials that demonstrated the non-inferiority of a fixed 120 mg SC dose compared to standard 5 mg/kg IV regimens [4,5,12], our findings confirm that this SC regimen provides adequate exposure, even in patients with intensified regimens. It should be noted that, in the database, only seven patients among all switchers exhibited low trough concentrations prior to changing to SC, which could be indicative of a loss of response. Furthermore, the low values of fecal calprotectin (Table 1) indicate a reduced risk of relapse and likely reflect the clinically stable disease status of many patients during maintenance therapy [5]. Therefore, the decision to change to the SC formulation was made based on its suitability for each patient.

According to our dataset, the SC-IFX trough levels observed are consistent with those reported in previous studies [12,18,19,20], and there are no differences between naïve and switch patients (Table 2). It was found that only 13% of patients required SC-IFX TDM-guided regimens. These subjects came from both the naïve and switch groups and had a weight lower than 80 kg. This low rate of TDM-guided adjustment should be interpreted with caution, as evidence supporting specific target trough concentrations and optimal dosing intervals for SC-IFX remains less well established than for IV-IFX [5,9,10]. This is because data specifically guiding proactive dose optimization for SC-IFX are still emerging [12,18,19,20]. Therefore, the limited number of SC-IFX regimen modifications in our cohort may partly reflect the absence of widely accepted SC-IFX-specific TDM thresholds and dosing algorithms, rather than a lack of potential need for optimization in selected patients.

The use of a PopPK approach, combined with the inclusion of switch patients, made it possible to quantify the impact of real-world patient-specific factors on the transition to subcutaneous IFX and drug exposure, providing a preliminary real-world framework for optimization guided by therapeutic concentration monitoring.

In view of the limited data available and the fact that all data were collected prior to the administration of the dose, the model demonstrates a superior fit with a one-compartment model with first-order absorption and elimination. The model under discussion is distinguished by its contrast with the bi-compartment model, which was developed using an intensive monitoring database of other published models [12,18,19]. This characteristic of the database could also be reflected in GOF (Figure 1). While the model’s performance is accurate for individual predictions, a slight bias can be observed at concentrations higher than 20 mg/L when considering population-level outcomes. Notwithstanding this, the model remains very useful for adjusting dosing via TDM in cases where a priori information is already available.

Covariate analysis identified TBW and SUSP as the primary determinants of SC-IFX CL. The inclusion of these factors resulted in a 10.1% reduction in the IIV of CL, ensuring that the CL variability remained consistent with the real one (25.7%). The RSE of 15.4% indicates adequate precision of the IIV estimate (Table 3). These findings support the relevance of the identified covariates in explaining between-subject variability in CL and suggest their potential utility for dose individualization.

Given the complexity of monoclonal antibody disposition, identifying predictors that explain such a substantial portion of IIV is crucial for clinical translation and personalized dosing strategies. However, given the relatively low proportion of patients with immunogenicity (26.7% of patients and 7.2% of samples), the CL_SUPS estimate should be interpreted with caution.

Conversely, none of the other measures of disease activity (CRP, FCP, ALB, HB, or FER) or demographic and clinical characteristics (AGE, SEX, HGT, BMI, ADM, DIAG, CREA, BIO, IMM, or STAT) exert a statistically significant or clinically relevant effect on the PK parameters in this cohort. While low albumin is a well-known driver of IV-IFX clearance due to its impact on the FcRn salvage pathway [12], its lack of significance here may reflect the relatively stable clinical status of our maintenance-phase cohort, where the “sink effect” of severe inflammation is less pronounced than during induction [4]. Although some PopPK models for IV-IFX include sex as a covariate [21], no sex-related significant differences were observed in the predictive performance of our model (Figure 1A,B). This is likely to be a result of the lower percentage of women in our population (31.1%). However, as illustrated by GOF and VPC (Figure 1 and Figure 2), female PREDs are generally worse than male PREDs.

Our model quantifies a substantial clinical impact for both predictors. Clearance was predicted to increase by 46.1% in a patient weighing 120 kg compared to a 70 kg individual. In a typical patient with a median TBW (73 kg), SUSP-positive concentrations were associated with a 70.0% higher CL compared with SUSP-negative observations. This estimate should be interpreted cautiously, as SUSP was defined as an exploratory composite covariate incorporating both confirmed ADA positivity and unexplained PK patterns suggestive of immunogenicity, rather than as the isolated causal effect of analytically confirmed ADAs. These findings align with those of previous SC-IFX PopPK models [12,19], which consistently identify body weight as a primary driver of IFX disposition. Similarly, Wang et al. [18] accounted for body size through allometric scaling, further supporting the biological plausibility of weight-based considerations, even in fixed-dose SC regimens.

A critical challenge in real-world TDM is the high rate of false-negative ADA results associated with drug-sensitive ELISA assays, which may underestimate immunogenicity in the presence of circulating drugs [20,22,23]. Furthermore, this analysis is not always performed as part of routine clinical practice. In order to address these limitations, and taking into account the repeatedly reported increased clearance in the presence of ADAs in IFX PopPK models [24], the observation–concentration SUSP covariate was introduced as a pragmatic PK-informed surrogate marker. This approach is supported by the previous literature on anti-TNF PopPK. A comparable, unexplained decrease in serum concentrations was formerly included as a covariate on CL in a PopPK model of subcutaneous adalimumab [11]. Nevertheless, as concentration decline is part of the SUSP definition, there may be some degree of circularity. Therefore, it is recommended that this covariate be regarded as exploratory.

According to our results, despite the fact that a flat-dose regimen for SC-IFX generally provides higher and more stable exposure, it may not be the optimal approach for certain subgroups. These results align with those of the simulation studies by Wang et al. [10], which indicated that, although 120 mg SC Q2W provides equivalent exposure to most intensified IV regimens (up to 10 mg/kg Q8W or 5 mg/kg Q4W), patients on the highest IV intensities—such as 10 mg/kg every 4 weeks—may require further SC dose optimization (e.g., 120 mg weekly) to avoid underexposure [10], as evidenced by the cases of three of our patients.

Although recent data suggest that SC-IFX may exhibit reduced immunogenicity, SC biologics have historically been considered more immunogenic than IV formulations [25,26]. In this clinical landscape, TDM transcends its traditional role as a reactive tool for loss of response. Proactive TDM remains essential for optimizing drug exposure and ensuring treatment efficacy [6], thereby mitigating the risk of de novo immunogenicity and subsequent treatment failure [6,7,27]. This is especially critical given that subtherapeutic levels are strongly associated with the development of anti-drug antibodies, which, as our model demonstrates, can increase IFX clearance by over 60%. In a setting of limited subsequent biological options, using PopPK-based TDM to optimize SC-IFX exposure represents a key strategy to enhance treatment persistence and preserve future therapeutic options.

Regarding the study limitations, the primary constraint is the relatively small sample size. In addition, as the study was conducted in a real-world setting and was designed to minimize patient burden, PK sampling was mainly focused on C_min_. This approach reduced the need for further hospital visits or blood tests. It is therefore crucial to employ methodological strategies that are considered acceptable, such as specific structural assumptions or internal validation [17]. Furthermore, this characteristic of the database exerts a slight influence on the model’s predictability at high concentrations in the absence of prior information. However, the clinical complexity of everyday practice is reflected in the database, in contrast to the more streamlined approach of pivotal clinical trials.

Secondly, the sex distribution of the data reflects clinical practice during the study period and was not determined by predefined criteria. Because SUSP was partly defined using longitudinal IFX concentration changes, this covariate is not fully independent from the PK outcome. Therefore, some degree of circularity/endogeneity cannot be excluded, and the statistical significance and magnitude of the SUSP effect on CL should be interpreted cautiously. Accordingly, SUSP should be regarded as an exploratory PK-informed surrogate marker rather than as a validated independent predictor of immunogenicity-related clearance.

Nevertheless, it has some strengths that should be noted. PK evidence and population models remain limited, particularly in real-life scenarios that include intensified dosing regimens. Our study addresses this gap by developing the first PopPK model with European real-world data and naïve patients. This model incorporates key clinical variables that are straightforward to quantify and that support personalized treatment strategies.

Future research should prioritize validating the model in external cohorts and simulating concentrations of dosage regimens based on model-informed precision dosing (MIPD) with this PopPK. Furthermore, it would be worthwhile to evaluate exposure–response relationships, with particular attention to the role of AUC versus C_min_ as pharmacodynamic predictors. Further investigations could help refine dose adjustments in patients with high body weight, with suspected immunogenicity, or transitioning from IV to SC formulations.

## 5. Conclusions

In conclusion, an exploratory population pharmacokinetic model was developed based on a modest real-world cohort of IBD patients. It was internally validated for SC-IFX, effectively capturing the drug’s disposition in a diverse (naïve and switch) cohort. Our findings identify TBW and SUSP as primary drivers of clearance, providing a pharmacological rationale for personalized dosing. This exploratory model has the potential to serve as a basis for future developments in MIPD, providing a practical framework for clinical transition toward more appropriate, self-administered biological therapies without compromising therapeutic precision. Ultimately, these results reduce the pharmacokinetic uncertainty surrounding subcutaneous infliximab in clinical routine, supporting its role as a precise and effective maintenance strategy for a broad spectrum of patients with IBD.

## Figures and Tables

**Table 1:** Patients’ baseline characteristics and median laboratory values.

Variable	Population
Patients (*n*)	45
Samples (*n*)	250
Age (years)	56 (38–61)
Total body weight (kg)	74 (65–82)
Height (m)	1.70 (1.60–1.72)
Body mass index (kg/m^2^)	26.1 (24.4–32.3)
Fecal calprotectin (μg/g) †	69 (00–171)
Creatinine (mg/dL) †	0.85 (0.77–1.01)
Serum albumin (g/dL) †	4.39 (3.96–4.78)
Ferritin (ng/mL) †	88.0 (39.0–147)
Hemoglobin (g/dL) †	15.1 (14.3–15.8)
C-reactive protein (mg/L) †	1 (0–2)
Sex (*n* (%))	
Male	31 (68.9)
Female	14 (31.1)
Treatment type (*n* (%))	
Naïve	16 (35.5)
Switch	29 (64.5)
Diagnosis (*n* (%))	
Crohn’s disease	16 (35.6)
Ulcerative colitis	29 (64.4)
Previous treatment with a biological drug (*n* (%))	10 (22.2)
Concomitant use of immunosuppression with azathioprine or methotrexate (IMM) (*n* (%))	30 (66.7)
Presence of anti-drug antibodies (ADAs) (*n* (%))	3 (6.67)
Suspected immunogenicity (SUSP) (*n* (%))	12 (26.7)

All continuous covariates are expressed as median (IQR, interquartile range Q1–Q3). Categorical covariates are expressed as absolute value (proportion), *n* = 45. Variables marked with † correspond to biomarkers with longitudinal measurements; therefore, the reported value represents the median of all samples collected (*n* = 250) from the 45 patients. For the remaining variables, values correspond to a single measurement per patient.

**Table 2:** Observed C_min_ of infliximab in steady state.

	IV-IFX Post-SC (Naïve) ^†^	SC-IFX Naïve	IV-IFX Pre-Switch	SC-IFX Switch
Mean (SD)	22.8 (12.3)	13.9 (4.87)	9.99 (5.81)	14.8 (9.17)

^†^ Post-induction IV-IFX concentration obtained before SC initiation (not a steady-state trough concentration).

**Table 3:** Population parameters for the basic model, final model, and bootstrap (*n* = 1000).

	Basic Model		Final Model		Bootstrap		Relative Error
Parameter	Value (Shrinkage)	RSE (%)	Value (Shrinkage)	RSE (%)	Median	CI 95%	(%)
Ka (h^−1^)	0.0113 *	—	0.0113 *	—	0.0113 *	—	—
CL (L/h)	0.0171	9.9	0.0170	8.8	0.0170	0.01433–0.02056	−0.1
V (L)	8.68	15.1	9.14	14.8	9.14	7.10–12.60	0.0
F_SC	0.698	9.9	0.750	10.0	0.767	0.631–0.938	2.2
CL_TBW_	—		0.00888	21.4	0.00870	0.00498–0.0137	−2.0
CL_SUSP_	—		0.700	43.1	0.737	0.465–1.93	5.3
IIV_CL (%)	28.6 (9%)	14.0	25.7 (8%)	15.4	25.0	16.9–32.9	−2.8
IIV_V (%)	0 *	-	0 *	—	0 *	—	—
IIV_F_SC (%)	28.4 (30%)	28.5	0 *	—	0 *	—	—
RUV (%)	38.5 (9%)	7.0	39.1 (6%)	7.4	38.6	32.9–44.1	−1.2

Abbreviations: CI, confidence interval; CL, clearance; CL_SUSP_, influence of suspected immunogenicity on CL expressed as a proportion; CL_TBW_, influence of total body weight on CL expressed as a proportion; F_SC, subcutaneous bioavailability; IIV, interindividual variability; Ka, first-order absorption rate constant; RSE, relative standard error; RUV, residual unexplained variability; V, volume of distribution. * Fixed values.

## Data Availability

The data that support the findings of this study are available on request from the corresponding author. The data are not publicly available due to privacy or ethical restrictions of Zenodo.org (https://doi.org/10.5281/zenodo.16040432).

## Supplementary Materials

The following supporting information can be downloaded at: https://www.mdpi.com/article/10.3390/pharmaceutics18091162/s1, Table S1. Sensitivity analysis of the fixed absorption rate constant (Ka) in the final population pharmacokinetic model, and Figure S1. Predicted steady-state infliximab concentration–time profiles according to total body weight following subcutaneous administration of 120 mg every 2 weeks.
