# Supplementary material for: Population Pharmacokinetics of Subcutaneous Infliximab: A Real-World Study of Naïve and Switch Patients
Source: Pharmaceutics. 2026 Sep 15;18(9):1162. doi: 10.3390/pharmaceutics18091162 (PMC13610694; doi:10.3390/pharmaceutics18091162)
Supplement: Supplementary file 1 [file pharmaceutics-18-01162-s001.zip › pharmaceutics-4512109-supplementary.pdf]

Population pharmacokinetics of subcutaneous infliximab: A real-world study of naïve and switch patients

Table S1. Sensitivity analysis of the fixed absorption rate constant (Ka) in the final population pharmacokinetic model.

**Model**	**Ka (h^{-1})**	**CL (L/h) (RSE %)**	**V (L) (RSE %)**	**F_SC (RSE %)**	CL_{TBW} (RSE %)	CL_{SUSP} (RSE %)	**IIV_CL(%) (RSE %)**	**RUV(%) (RSE %)**
Ka1	0.00565	0.0168 (8.3)	8.93 (14.1)	0.696 (9.3)	0.00916 (21.1)	0.664 (40.4)	26.2 (15.6)	38.9 (7.5)
Ka2	0.00848	0.0169 (8.9)	9.06 (15.1)	0.726 (9.7)	0.00900 (21.2)	0.686 (42.0)	25.9 (15.5)	39.0 (7.5)
**Hanzel et al Ka**	**0.01130**	**0.0170 (8.8)**	**9.14 (14.8)**	**0.750 (10.0)**	**0.00888 (21.4)**	**0.700 (43.1)**	**25.7 (15.4)**	**39.1 (7.4)**
Ka3	0.01410	0.0170 (8.4)	9.19 (14.1)	0.770 (9.1)	0.00880 (21.5)	0.709 (41.5)	25.5 (15.4)	39.2 (7.3)
Ka4	0.01690	0.0170 (8.8)	9.21 (14.5)	0.786 (9.4)	0.00873 (21.5)	0.714 (40.9)	25.3 (15.4)	39.2 (7.3)

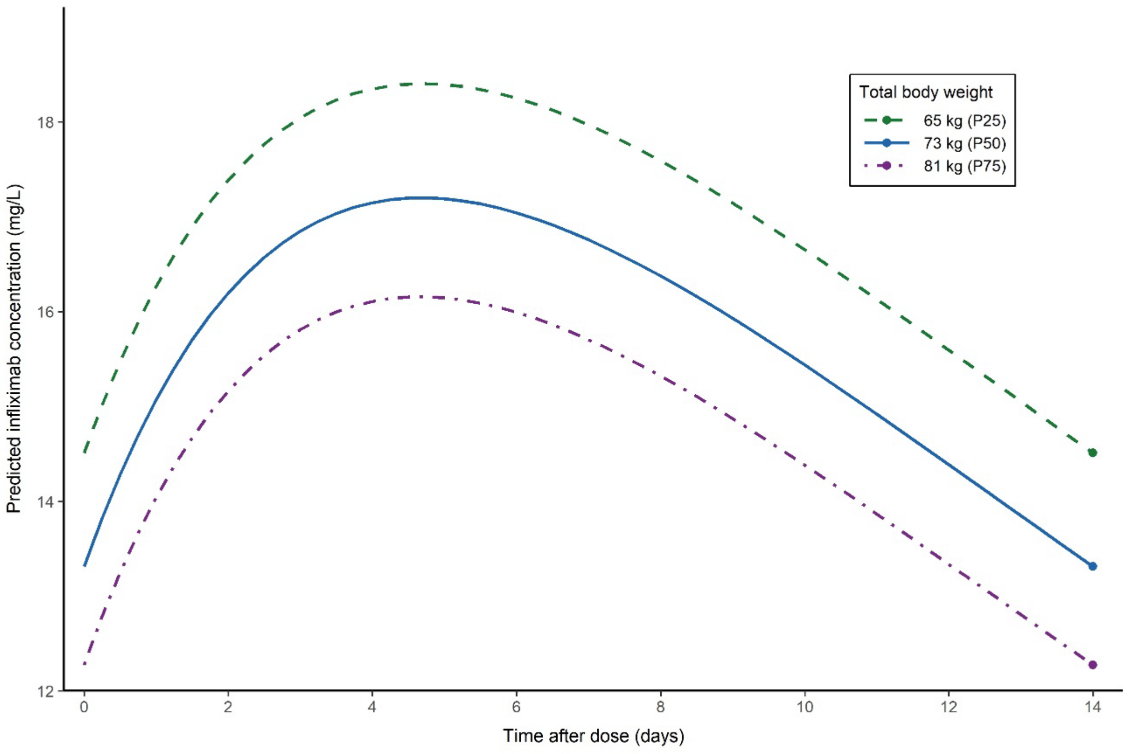

Figure S1. Predicted steady-state infliximab concentration–time profiles according to total body weight following subcutaneous administration of 120 mg every 2 weeks.
